# Lectures on Diseases of the Skin

**Published:** 1886-08

**Authors:** Henry Wile

**Affiliations:** Lecturer on Dermatology, Atlanta, Ga.


### LECTURES ON DISEASES OF THE SKIN.

DELIVERED AT THE ATLANTA MEDICAL COLLEGE DURING THE SESSION OF 1885–6, BY HENRY WILE, M. D., LECTURER ON DERMATOLOGY, ATLANTA, GA.

### LECTURE VI.

GENERAL DIAGNOSIS—GENERAL CONSIDERATIONS—WITH REFERENCE TO THE PATIENT—WITH REFERENCE TO THE DISEASE—GENERAL PROGNOSIS.

Reported Stenographically for the ATLANTA MEDICAL AND SURGICAL JOURNAL by W. Kay Tewksbury.

The differentiation of diseases is a subject of special importance in practical medicine. The diagnosis determines the prognosis and treatment and is therefore a matter for the satisfaction of the physician and the welfare of the patient. In cutaneous medicine, where the symptoms are in the main objective, where little reliance can be placed on the statements of the patient, where sight and touch must appreciate and judgment alone interpret, the subject of diagnosis offers some difficulty. To the student or beginner diseases of the integument at first present themselves with details so complex, and a similarity so striking, as to make the matter of diagnosis appear arbitrary, and, in fact, the whole subject of dermatology seem a mass of confused nomenclature. But this is different when certain methods of observation are pursued, certain general considerations are kept in mind and appreciated, and a proper clinical conception of cutaneous disease is formed. In arriving at a diagnosis, the mind, in each case, goes through a certain mental process, and the diagnosis is reached by a species of exclusion. When the eruption is characteristic and of common occurrence, the mental process is a quick one and the diagnosis is made at once. On the other hand, when the disease is rare and presents complications, the process is slow, and some time is required for study before a diagnosis is reached.

In the first place the examination should always be conducted according to method. One of the first objects being to gain the confidence of the patient, you may inquire in a general way as to the disease, its duration, extent, mode of development. This is done, not with a view of acquiring reliable information upon which to base a diagnosis, but simply as an appropriate manner of introduction and preface to the further investigation of the case. Not infrequently the patient will begin a recital of observations made at different times and volunteer information to which the physician will necessarily lend a respectful ear. But such knowledge must be received with great reserve. The history of the case, as learned from the patient, is usually to be ignored in making a diagnosis. It is seldom satisfactory, generally obscure and incomplete, and often misleading. This is due to the ignorance or embarrassment of the patient, or to a desire to conceal unfortunate private affairs. Thus, in a majority of cases of syphilitic eruptions of the skin, the patient will dissemble, deny point-blank or affect ignorance. The diagnosis must finally depend upon the judgment of the physician and his ability to interpret the characters of the disease as they appear upon the integument. The history of the case must be reconciled to the diagnosis, not the diagnosis to the history. It may at most be suggestive. Thus when the statement is made that the disease is most troublesome at night just after going to bed—that the itching is then intense—it is suggestive of scabies.

In making an examination it should be borne in mind that a good light, preferably reflected sunlight, and where much of the surface of the body is exposed a proper temperature are desirable. Color differences may be slight between normal and diseased skin, so that by a poor or artificial light, lesions might escape detection. Then again the proper determination of the color of lesions may reveal the stage of the process, whether recent or at the point of absorption. A good light may also bring out more sharply certain minor features, as slight elevation, desquamation, etc. The skin is susceptible to sudden temperature changes, therefore when a large part of the surface of the body is to be exposed, the temperature of the room should be at a proper degree.

If the eruption be diffuse, the whole surface of the body should be examined, for it is exceedingly important to get a view of the disease in its entirety, as no reliance is to be placed upon statements of patients with reference to lesions that are concealed by the clothing. In making this examination, the eye of the physician must take in all the general and special features of the disease, together with any unusual conditions of the skin in general, such as a surplus or want of subcutaneous fat, pigment, blood, hair, etc. By means of the fingers, consistence, depth and existence of infiltration may be determined; also the difference between hyperæmia and hemorrhage or pigment—the former disappearing on pressure, the latter remaining.

A valuable aid in diagnosis is often found in the microscope. This instrument furnishes information as to the histology of the lesions, and if the disease is of a parasitic nature, the parasite can be detected and the diagnosis at once established. In cases of doubt it should always be resorted to. Chemical analysis at times offers data that shed some light on the nature of the disease. Thus the chemical examination of the urine may reveal sugar, indicating diabetes mellitus, or albumen, Bright's disease, which diseases are often associated with pruritus, the former especially with furunculosis and gangrene. Blood corpuscles in the urine may suggest purpura.

The other general considerations which ought to be taken into account in making a diagnosis may be divided as follows:

*1. Those having reference to the patient.*

2. *Those having reference to the disease.*

1. The causal relation existing between cutaneous diseases and systemic disturbance or disorders of other organs of the body has been referred to under the subject of general etiology. From what has been said it follows that a knowledge concerning the general health of the patient and the existence of any organic or functional disease is of value, and in certain instances such knowledge may furnish corroborative evidence as to the diagnosis. Thus the existence of fever or increase of pulse rate may indicate eruptive fever, as variola, varicella; rheumatism may give evidence of erythema, erythema multiforme; gastric or intestinal irritation, chronic erythema and urticaria.

The age of the patient should be noted, for it has already been pointed out that many diseases of the skin are peculiar to or identified with certain periods of life. Thus hereditary syphilis, urticaria to infancy; ichthyosis, impetigo contagiosa, tinea circinata to childhood; acne to youth; epithelioma to old age.

The same may be said of sex, temperament, habit, social condition and occupation. A certain amount of general information may be elicited from a careful inquiry as to these points, which may be useful in making the diagnosis, but it should be regarded only as corroborative evidence. Thus lupus erythematosus and pruritus occur more on females, whereas sycosis (barber's itch) is confined to males. Seborrhœa attacks blondes more frequently than brunettes. Erythema nodosum and erythema multiforme are generally associated with rheumatism, and chronic forms of eczema with gout. Intertrigo and hyperidrosis are especially encountered on corpulent individuals. Parasitic diseases, impetigo contagiosa and ecthyma are almost confined to the poorer classes. Eczema fissum is common among washwomen, and acne among workers in oils.

2. With reference to the disease, it should be observed whether the lesions are single or multiple, symmetrical or asymmetrical. Then its symptomology is to be determined; the lesions being closely inspected, their exact nature is established, whether macular, papular, vesicular, pustular. The disease may be characterized by one form, as in lichen ruber, acne, eczema by the papule; sycosis non-parasitica by the pustule, or it may be polymorphous as in scabies and syphilis, where macules, papules, vesicles and pustules may all be present at the same time.

If the disease appears in a macular form the spots may be on the surface of the skin, or in the most superficial layers, as in tinea versicolor; or in the lower layers, due to the presence of deeply-seated lesions that are approaching the surface. The spots may be the result of hyperæmia, as in erythema; of hemorrhage, as in purpura hemorrhagica, or pigmentation, as in chloasma and lentigo.

If the eruption is papular, the papules may be variously shaped and sized—as a rule pin-head sized and rounded in eczema, while in papular syphiloderm they may be as large as a pea and flattened. They may appear and disappear as papules, in lichen ruber, or develop into vesicles, in eczema and variola. A papule may be the result of inflammation, as in papular eczema or acne, or due to a collection of epidermal scales around the outlet of the hair follicles as in lichen pilaris.

Vesicles are recognized by their watery contents. They appear at once in varicella, vesicular eczema and herpes zoster, or as a further development of papules, as in eczema and herpes iris. They may show a tendency to group and remain intact, as in all herpetic eruptions, or to appear scattered and soon rupture, as in vesicular eczema. Sometimes they show a disposition to coalesce, forming larger lesions as in herpes zoster.

Blebs may result from injury, such as a scald or application of vesicants, or may be characteristic of such diseases as erysipelas and pemphigus. In the former case the lesions are confined to the locality injured and are divided into compartments, while in erysipelas the local inflammatory symptoms are marked, and there is, besides, attending constitutional disturbance. In pemphigus the blebs are extremely superficial and one-chambered.

Wheals are lesions that indicate an over-sensitiveness of the skin. When single they may be the result of the stings of insects, such as the mosquito or bedbug. Frequently they appear reflexly as the result of gastric or intestinal irritation, or of indulgence in certain articles of diet, as in urticaria.

Pustules appear as a later stage of development of the papule or vesicle, as in acne, variola, herpes zoster, eczema and pustular syphiloderm, or they appear at once as pustules, as in impetigo, ecthyma and sycosis non-parasitica. Their location is often characteristic of the disease. Thus when confined to the scalp of children, pustular eczema is indicated; when the non-hairy part of the face of young persons is affected, it may in a majority of cases be looked upon as acne; when the hairy part of the face, sycosis non-parasitica. Universal pustulation, in which the lesions are scattered over large areas, suggests variola, varicella or pustular syphiloderm.

Tubercles are characteristic of sycosis (parasitic) when confined to the bearded part of the face. The same, when localized on other parts of the body, arranged in semi-circular or crescentic form and exhibiting a disposition to ulcerate, are most commonly syphilitic in character.

Excoriations, as already noted, form a valuable diagnostic sign indicating sensory nerve irritation and the existence of itching. The depth of the excoriation shows the intensity or degree of nerve irritation; the distribution corresponds to the extent or location of the disease; whereas the number and existence in different stages of the process of healing may give some idea as to the duration of the affection.

Scales present characteristics that are peculiar to certain diseases; thus they are fine and dry in tinea circinata; oily, small in seborrhœa; thin, large in eczema; lamellated, pearly in psoriasis; plate-like in ichthyosis.

Crusts offer, at times, some diagnostic hints. When light-colored, or composed of serous exudation, they generally indicate a superficial process, as eczema, while those of a dark color, showing an admixture of blood, denote a process more deeply seated, as ecthyma. Thick, lamellated, conical, "oyster-shell shape" crusts are peculiar to syphilitic rupia, while round, cup-shaped, small pea-sized, yellowish, friable crusts are characteristic of tinea favosa.

Fissures at the border of skin and mucous membrane, as around the mouth and anus, especially in children, are always to be regarded with suspicion of syphilis, while those occurring on the fingers, especially during cold seasons, are usually eczematous.

Ulcerations indicate a destructive process, and are the results of foregoing disease. They generally bear upon them the impress of the preceding disease; thus syphilitic ulcerations present sharply-defined, indurated borders, and, as a rule, assume a crescentic or serpiginous form so peculiar to syphilitic eruptions.

Scars present the positive diagnostic sign that there had existed some inflammatory process attended with loss of tissue. The size and number of scars show the extent of the disease. Those resulting from syphilitic ulcerations are local. Scalds and mechanical injuries may also give rise to local scars, which are, as a rule, more or less irregular and contracted. Linear scars on the abdomen and thighs of a female denote former distension of the abdominal cavity from pregnancy or disease. In pustular diseases, like variola, acne atrophica, herpes zoster, the scars are small and scattered.

Pigmentation is likewise the residue of disease. When diffuse it may be symptomatic of internal disease, as Addison's disease, liver and uterine disorders, or due to continuous internal use of nitrate of silver (argyria). When seen in spots it may be the result of foregoing hemorrhages, purpura, infiltrations, syphilis, lichen ruber, or due to a deposit of true pigment, as in lentigo (freckle) and pigment nævus. When limited to certain localities it may suggest some long-continued irritation; thus, limited to the region of the shoulders and hips, it is often associated with pediculosis.

It may occur that the crusts or scales are so abundant as to cover up the true lesions and their characteristics. This is often the case in diseases attacking the scalp, as eczema, psoriasis and seborrhœa, also in certain forms of ulcerations, as epithelioma and syphilis. In making a differential diagnosis between these processes, it is at times important to get a view of the lesion proper, and when the crusts or scales are adherent, it is necessary to soften and remove them.

It should always be determined whether a disease is acute, chronic, or appears in the form of a relapse. The first are usually more or less diffuse or cover a large surface, as the exanthemata, the eruptions of early syphilis and medicinal eruptions. Chronic diseases of the skin are, as a rule, confined to some locality; thus chronic forms of eczema, acne, epithelioma, late syphilitic manifestations. Some diseases often disappear spontaneously or after proper treatment, but are liable to recur at any time, such as psoriasis, sycosis non-parasitica, acne, rosacea, eczema and epithelioma.

The fact that diseases of the skin exhibit a predilection for certain localities should be remembered. Particularly is this the case with parasitic affections; thus scabies first attacks the interdigital spaces and flexor surfaces, bend of the elbow and axilla; pediculosis is always most marked in those parts against which the clothing rests closely, as about the shoulders and hips; tinea sycosis is confined to the bearded part of the face; tinea versicolor to the chest and back. Of other diseases epithelioma occurs mostly on the face, around the nose, eyes, mouth and ears; acne, rosacea and comedo on the face, the first occasionally on the chest and back; seborrhœa on the scalp. Finally, in regard to the localization of diseases, it may be observed that the early eruptions of syphilis are generally more marked on the flexor surfaces. On the other hand, diseases of a more superficial type, especially those of the epidermis, which are of a more or less desquamative nature, occur more on the extensor surfaces of the body, as psoriasis, ichthyosis, eczema. The color of the lesions is peculiar in some diseases; thus in herpes iris there is a variegated or rainbow appearance; syphilitic papules and tubercles are usually of a ham or coppery color.

The subjective symptom of itching must always be taken into consideration as a diagnostic point. It invariably accompanies eczema, but is rarely present in syphilitic diseases. All parasitic affections and most of the acute exanthemata are characterized by its presence, while the cutaneous diseases resulting from atrophy and hypertrophy occur, as a rule, without it. Itching, when marked, is always to be recognized by excoriations. These may be so extensive as to cover up the lesions of the disease proper, as in neglected cases of scabies, or to modify them, as in acute eczema.

These mechanical additions to the symptoms of disease must be eliminated in the diagnosis, yet at the same time the subjective symptom is to be taken into account. The same may be said of neuralgic pains, as in herpes zoster, prickling in urticaria and miliaria, and burning in dermatitis.

The most important matter, perhaps, in the formation of the diagnosis is the study of individual lesions in such a manner as to trace correctly the mode of development and course of the disease. It should be determined whether the lesions are superficial or deeply seated—whether circumscribed, sharply defined, not affecting the surrounding tissue, or whether they are diffuse, having a tendency to spread peripherally. Thus some lesions cease to develop after attaining a certain size, as those of acne, herpes zoster, and the exanthemata; they disappear by absorption, whereas the ulcerations of syphilis and epithelioma have a disposition to increase indefinitely without interference. The course which a disease runs is a most valuable matter as an aid in the diagnosis, as each disease of the skin runs its peculiar course, and has its peculiar mode of development.

General Prognosis.—A knowledge as to the course, termination and curability of cutaneous diseases is always necessary in the successful practice of dermatology. The inquiries of the patient are, as a rule, first directed to this subject, and the skill as well as the judgment of the physician are very often rated according to the results of his predictions. The prognosis of diseases of the skin is in general more favorable than that of diseases of other organs of the body. Although most cutaneous affections are inclined to run a chronic course, many are disposed to relapse and some to end fatally, there are none in which the symptoms cannot be at least ameliorated, not even desperate cases in which life cannot be prolonged.

A correct prognosis depends upon a correct diagnosis, and its verification, in a great measure, upon the ability to give the appropriate treatment. In most acute diseases, especially those attended with constitutional disturbance, as the exanthemata, the erythemata, dermatitis medicamentosa and venenata, the prognosis is favorable. In diseases that are inclined to run a chronic course, as eczema, acne, in those that are likely to recur, as psoriasis, syphilis, epithelioma, although much can be accomplished in the way of treatment, it is well, as a rule, to offer a guarded prognosis. In case of parasitic diseases, a cure may always be promised with security.

